# Enhanced Performance of Camphorsulfonic Acid-Doped Perovskite Solar Cells

**DOI:** 10.3390/molecules27227850

**Published:** 2022-11-14

**Authors:** Adam Wincukiewicz, Ewelina Wierzyńska, Aliaksei Bohdan, Mateusz Tokarczyk, Krzysztof P. Korona, Magdalena Skompska, Maria Kamińska

**Affiliations:** 1 Faculty of Physics, University of Warsaw, Pasteur 5, 02-093 Warsaw, Poland; 2 Faculty of Chemistry, University of Warsaw, Pasteur 1, 02-093 Warsaw, Poland

**Keywords:** perovskite, photovoltaics, perovskite solar cells

## Abstract

High-quality perovskite film with large grains and therefore reduced grain boundaries plays a significant role in improving the power conversion efficiency (PCE) and ensuring good long-term stability of the perovskite solar cells. In this work, we found that adding camphorsulfonic acid (CSA), a Lewis base, to the perovskite solution results in the crystallization of larger perovskite grains. By varying the concentration of CSA, we found that the optimal concentration of the additive is 1 mg/mL, which leads to an 20% increase in PCE of the cells compared to the reference CSA-free cell. Interestingly, we observed that the PCE of cells with an excess of CSA was initially poor, but may increase significantly over time, possibly due to CSA migration to the hole-transporting layer, leading to an improvement in its conductivity.

## 1. Introduction

Hybrid organic–inorganic perovskites have many advantages for application as an active layer in solar cells, such as high absorption in the visible range, extended diffusion length of free carriers, their high mobility, and long recombination lifetime [1]. Despite these excellent optoelectronic properties, the morphology of deposited perovskite layers leaves much to be desired and they remain sensitive to diffusion of harmful environmental components, so under normal conditions, they degrade fast. Likewise, perovskite solar cells (PSCs), regardless of some protection of the active layer by the cell structure itself, suffer from major stability problems and are sensitive to external factors such as oxygen, moisture, overheating, or UV illumination [2,3]. To shield PSCs from extrinsic agents, it is necessary to perform all steps of solar cell preparation in a protective atmosphere and finally encapsulate the device. One of the approaches to improve the intrinsic properties of perovskites that affects the stabilization of solar cell efficiency and electrical parameters is the use of special additives to the perovskite [4,5]. In the literature, a wide variety of admixtures such as acids, salts, polymers, and nanoparticles [6] are mentioned. They all produce a similar effect in terms of changing the perovskite morphology. Namely, they stimulate the processes of nucleation and crystallization of the perovskite in the form of large grains. Moreover, the molecules of additives localize in the grain boundaries (GBs), passivating the possible trap sites. Additionally, some of them, such as polymers, act as the charge transport materials in the interfacial layers, improving the charge separation and hindering the recombination process [7].

According to the literature, the addition of HCl [8] or HBr [9] acids affects the morphology of the perovskite layer. These acids improve the solubility of precursors and reduce the size of colloid particles. Moreover, during the evaporation of the solvent from a mixture of PbI_2_ and methylammonium iodide (MAI), the crystallization process is slower, which results in the formation of larger grains [6]. Moreover, an organic acid with a carboxylic group has been tried as additive during the synthesis of the perovskite. It has been found that ascorbic acid (AA) added to Pb/Sn-binary perovskite modifies the process of its crystallization, affecting the morphology and therefore inhibiting the aging process of the perovskite. Solar cells with AA have long photogenerated carrier lifetimes, ensuring PCE growth from 12.18 to 14.01% [10]. Su et al. introduced trimesic acid (TMA) to the precursor solution, which enhanced PCE from 14 to about 17%. The TMA additive plays a meaningful role in the morphological changes, the crystalline structure of perovskite, and stability in the air. It has been suggested that the aromatic structure of the additive and hydrogen bonding between the acid and iodine atom from the perovskite suppress an ion migration in the active layer [11]. Another kind of additive incorporated into the perovskites are organic acids containing sulfonic groups, such as 4-methylbenzenesulfonic acid (4-MSA) [12]. Similarly, it improves crystallinity and morphology and reduces hysteresis due to fewer grain boundaries and an enlarged grain size.

In our previous study on the perovskite and polymer-based solar cells, we introduced the polyaniline doped with camphorsulfonic acid (CSA) as a hole-transport layer (HTL) [13]. The solar cells with HTL with an excess of CSA gained better PCE in a long-time scale. We suppose that CSA may be located at the interface of HTL and active layer or migrate to the active layer. Therefore, the aim of this work was to investigate the influence of the addition of CSA directly to the perovskite precursor on the morphology and crystallinity of the perovskite and the efficiency of the resulting solar cell.

## 2. Results and Discussion

### 2.1. The Influence of Camphorsulfonic Acid (CSA) on the Perovskite Structure

**Figure 1:**
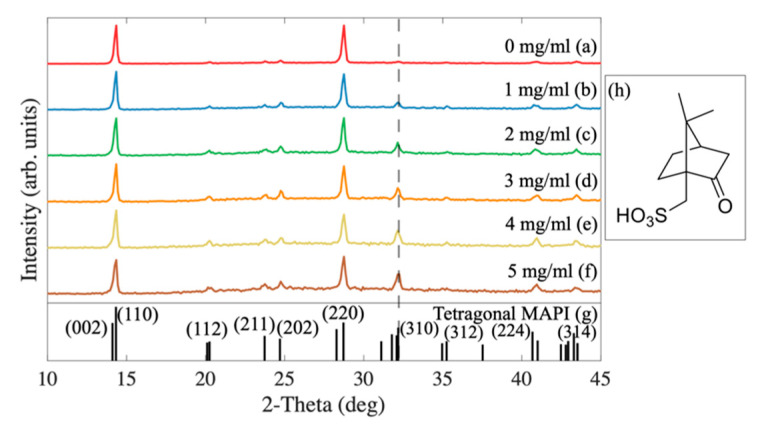
XRD patterns (Cu K-alpha radiation) of MAPI with different amounts of CSA (**a**–**f**), reference derived from JCPDS No. 01-084-7607 (**g**). Dashed line represents a sample diffraction pattern, in which contribution increases with CSA content. (**h**) CSA molecule.

The XRD patterns of the fabricated perovskite films with different amounts of CSA (Figure 1h) are shown in Figure 1. They all reveal most of the diffraction peaks characteristic for the perovskite structure and indicate the highly polycrystalline morphology of the films, with close to (but not full) random grain orientation. The dominance of (110) and (220) reflexes indicates the partial texturization of the solidified perovskites. There is no evidence of additional phases such as PbI_2_ or CSA.

**Figure 2:**
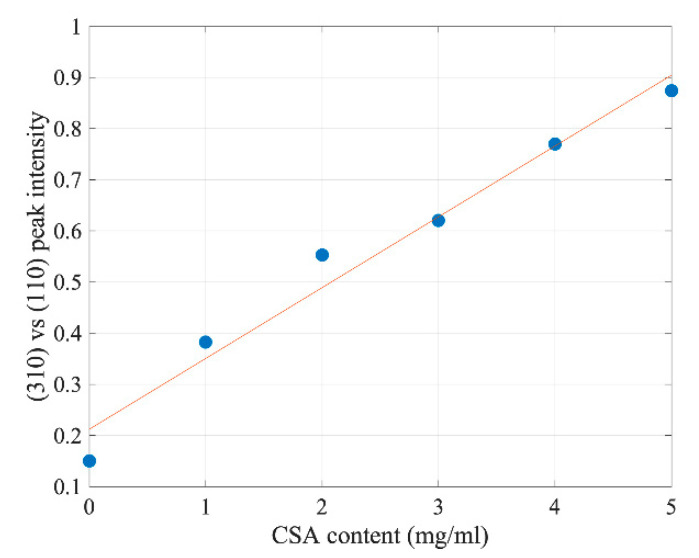
Peak intensity of (310) vs. (110) as a function of CSA content within MAPI precursors.

Interestingly, the higher the concentration of CSA within the perovskite, the stronger the additional peaks were compared to the main (110) and (220) diffraction peak intensities, such as the exemplar (310) peak marked with a dashed line in Figure 1. Figure 2 shows its relative intensity to (110) diffraction peak intensity as a function of CSA content. The almost linear increase indicates a reduction of the degree of solidified perovskite texturization with increasing CSA amounts added to the precursor solution.

This result is evidence of the increasing randomization of grain orientation. Interestingly, it has been reported that additives such as 4-methylbenzenesulfonic acid (4-MSA) improve the perovskite’s crystallinity [12]. Due to its aromatic character, 4-MSA may intercalate the structure of perovskite, leading to materials’ growth according to the 4-MSA orientation within the film. On the contrary, the CSA used in our studies is not an aromatic compound; it contains a sulfonic -SO_3_^−^ group that may interact with Pb^2+^ ions. According to Liu et al. [14], the density of Pb atoms in the perovskite is the largest on (110) and (002) facets. Therefore, in the presence of the additive containing a sulfonic group (3-(decyldimethy- lammonio)-propane-sulfonate inner salt in [14]), the growth of these facets may be slowed down and result in higher randomization of crystallites growing in the presence of CSA. In general, from the point of view of the material electrical properties, texturization would be the preferred form of crystallization [15], as it should enhance the mobility of carriers along the plane of perovskite layer. For the perovskite solar cells, however, the mobility across the layer is important, so the observed texturization should not be of any significance for the operation of the cells.

**Figure 3:**
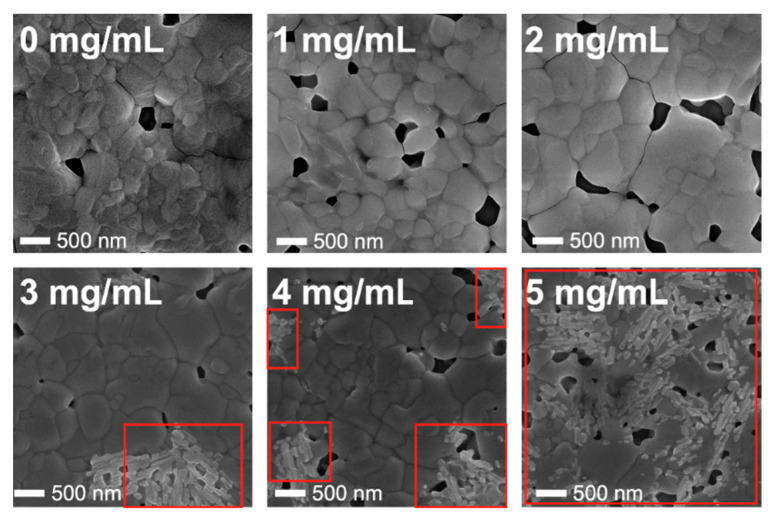
SEM images of MAPI: CSA samples. Areas with CSA crystallites are marked with red squares.

The SEM images of the obtained perovskites with different CSA contents are presented in Figure 3. It was found that for a relatively high concentration of CSA, the acid precipitations occurred on the surface of the samples. This was easy noticeable for the samples with CSA concentration equal to or higher than 3 mg/mL, and the areas with CSA crystallites are marked with red squares. The higher the CSA concentration, the greater the surface of perovskite covered with acid crystallites. Even though we did not observe any traces of CSA precipitation on perovskite films with no CSA (0 mg/mL) to 2 mg/mL, based on CSA properties (melting and boiling points of 196–200 °C and 344 °C [16], respectively), we suppose that it remains dissolved in the film (perovskites are annealed at the significantly lower temperature of 100 °C).

**Figure 4:**
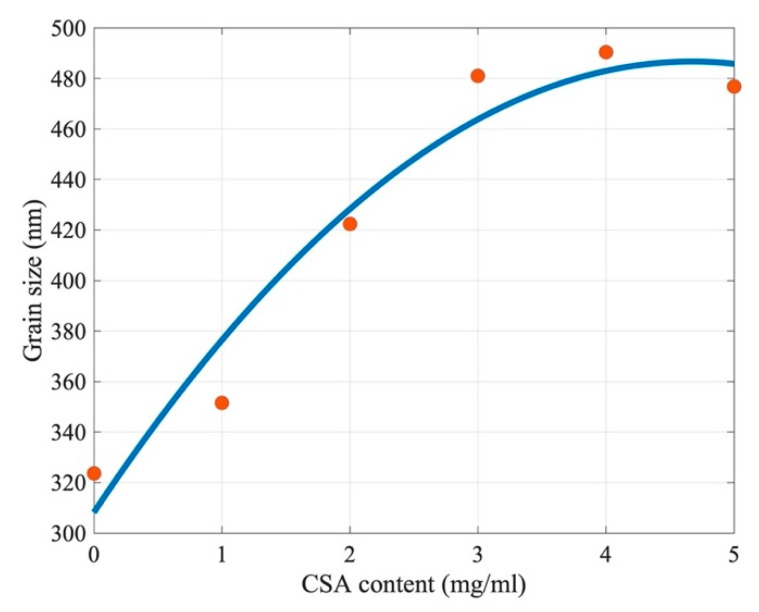
Perovskite grain sizes as a function of CSA content. The red dots are experimental data, and the solid line is a guide for the eye.

In Figure 3, one can also observe a gradual increase of the average grain size from 320 nm for perovskite with no CSA (0 mg/mL) up to 480 nm for perovskite with 3 mg/mL CSA. For the samples with higher CSA content (4 and 5 mg/mL), a plateau of grain sizes was reached at a level of about 480 nm (Figure 4).

According to the literature, the SO_3_^−^ groups of 4-MSA may interact with methylammonium ion (MA^+^) in the precursor solution, making a number of MAI surrounded by additives containing SO_3_^−^ [12]. During crystallization, the interaction between MAI and 4-MSA promotes the growth of large perovskite grains. The CSA used in our studies also contains SO_3_^−^ group; therefore, we suppose that the same mechanism is responsible for the increase of the perovskite grain size with increasing CSA concentration in the precursor solution.

On the other hand, PbI_2_ is likely to bond with Lewis bases [17]. It has been shown that the addition of DMSO (Lewis base) to DMF used as the solvent of perovskite precursors affects PbI_2_-DMF cluster formation on the molecular level because DMSO shows much stronger coordination capability to PbI_2_ than DMF [18]. Since CSA is also a Lewis base, having three oxygen atoms with lone electron pairs, it may interact even stronger with PbI_2_ than DMSO, leading to smaller colloid particles that act as nucleation centers during the perovskite formation.

Thus, we believe that both SO_3_^−^ group interactions with CH_3_NH_3_^+^ from MAI and Lewis acid–base interactions between Pb^2+^ from PbI_2_ and CSA lead to better dispersion of the perovskite precursor molecules, promoting the growth of the larger perovskite grains upon annealing compared to the those that grow without CSA. We also found that the optimum CSA concentration regarding the grain size was in the range of 3–4 mg/mL (Figure 4). However, it should be remembered that starting from a 3 mg/mL CSA content, the problem with CSA precipitation begins.

### 2.2. Photovoltaic Properties

**Figure 5:**
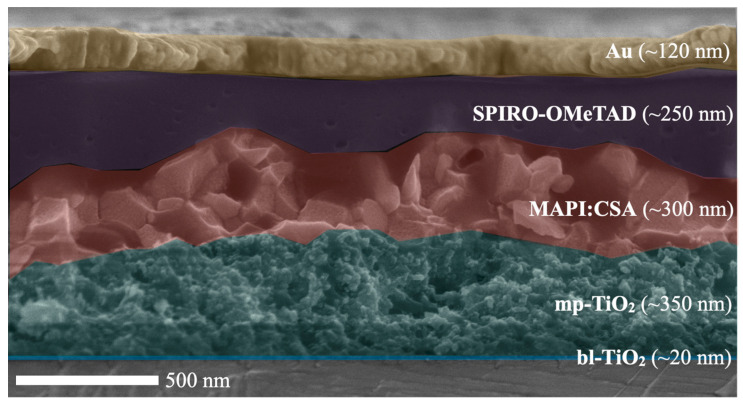
SEM image cross-section of perovskite solar cell.

Perovskite solar cells in the configuration ITO/TiO_2_/perovskite/HTL/Au were prepared according to the procedure described in Section 3.3. The SEM image of this device cross-section is shown in Figure 5. Due to fluctuating thicknesses of the layers, we show the average values in Figure 5 based on 20 measurements of the thickness of each layer.

In the studies, the perovskite doped with CSA concentrations ranging from 0 to 5 mg/mL was used as the active layer.

Current-voltage (J-V) characteristics of the constructed cells were measured using KEITHLEY 2450 Source Meter with Kickstart PC software, and the cells were attached to an Ossila 8-pixel test board during the measurements. They were illuminated by Newport VeraSol-2 LED Class AAA Solar Simulator of 1000 W/m^2^ power output and AM1.5G spectrum.

**Figure 6:**
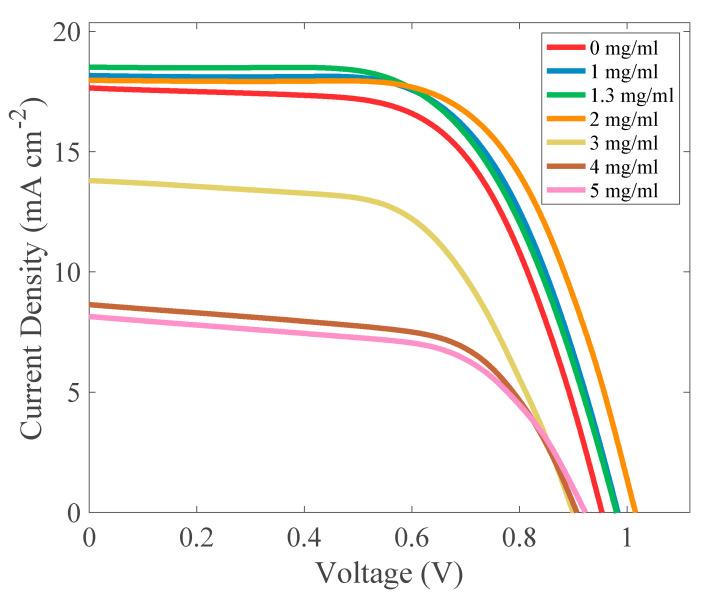
J-V characteristics of the perovskite solar cells with different CSA content.

Examples of the J-V characteristics of perovskite-based devices with different CSA concentrations are compared in Figure 6. As can be observed, all the cells revealed proper, diode-like characteristics with electrical parameters presented in Table 1. The most important parameter of the solar cell is its efficiency. As can be seen from Table 1, the addition of CSA to the active layer improved the performance of perovskite solar cells. The highest power conversion efficiency PCE = 11.73% was observed for the sample with a CSA concentration of 2 mg/mL. This cell had an open-circuit voltage of V_oc_ = 1.02 V, short-circuit current density of J_sc_ = 17.96 mA cm^−2^, surface serial resistivity of ρ_s_ = 7.03 Ω cm^2^, and surface shunt resistivity of ρ_sh_ = 5360.40 Ω cm^2^. However, the highest average PCE based on eight pixels of each device was obtained for the perovskite cell with a CSA concentration of 1 mg/mL. Table A1 in the Appendix A contains the average values of the photovoltaic parameters of the investigated set of solar cells.

**Figure 7:**
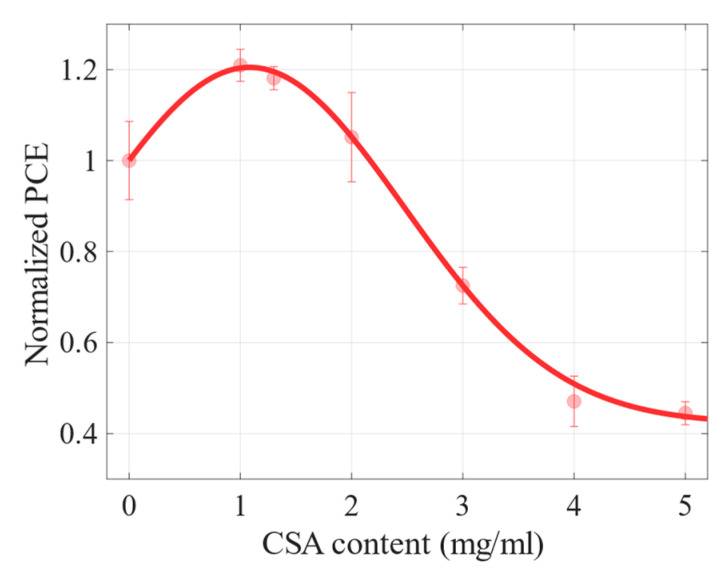
The normalized PCE vs. CSA content within perovskite precursors. Error bars represent a standard deviation. The red dots are experimental data, and the solid line is a guide for the eye.

The normalized efficiencies (meaning the efficiency of a particular cell divided by the efficiency of the reference (CSA-free) sample) of the cells are shown in Figure 7. A CSA concentration of 1 mg/mL within the perovskite precursor improved the PCE by 20% compared to the reference cell. A sample with 1.3 mg/mL of CSA was additionally fabricated to possibly maximize the PCE, but the device based on this perovskite had slightly lower efficiency than the one with 1 mg/mL, which indicates that concentration of 1 mg/mL is very close to the optimal content of the acid. Generally, the average efficiencies of the devices based on perovskites with CSA concentrations of up to 2 mg/mL were improved compared to the reference CSA-free cell. The increase in efficiency of perovskite cells with CSA was primarily the result of an increase in short-circuit current density and an improvement in open-circuit voltage, but also a significant increase in shunt resistance. The observed enlargement of the perovskite grain size undoubtedly contributed to the improvement of charge transport through the perovskite layer and especially led to higher mobility. Moreover, the reduced content of the grain boundaries most likely contributed to an increase in the shunt resistance and an improvement in the fill factor. It has already been shown that perovskite grain size affects PCE [19,20]. We suspect that bigger grains of perovskites with 1, 1.3, and 2 mg/mL of CSA had a positive impact on the performance of the cells based on these materials.

**Figure 8:**
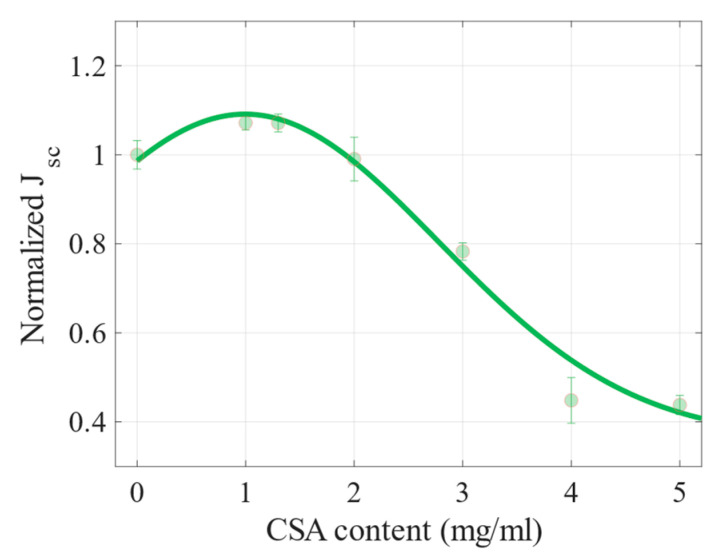
Normalized current density (J_sc_) vs. CSA content.

**Figure 9:**
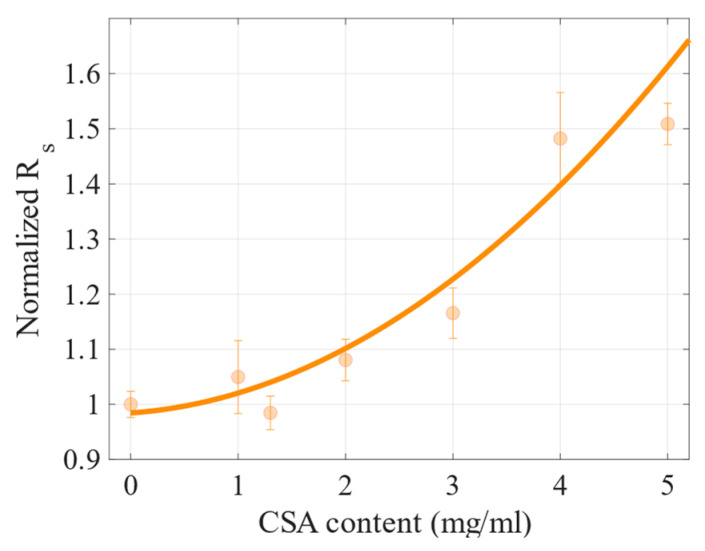
Normalized series resistance (R_s_) vs. CSA content.

**Figure A2:**
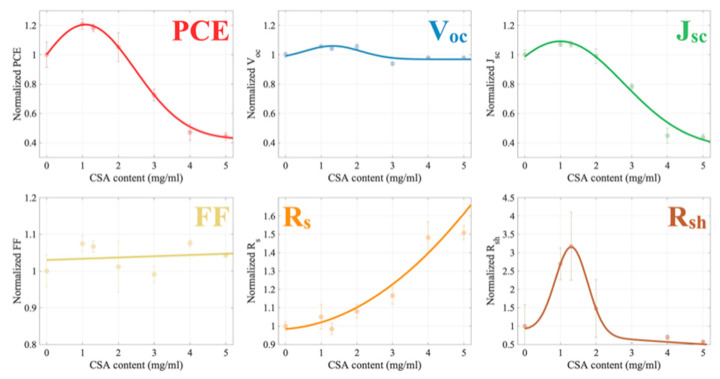
Photovoltaic parameters of the solar cells.

For the cells with 3, 4 and 5 mg/mL of CSA, the PCEs were lower than that of the reference device; particularly, the cells with the two highest acid concentrations retained only half of its performance. The two main parameters contributing to such a significant decrease in PCE were changes in the current density decreasing (Figure 8) and series resistance increasing (Figure 9). Both figures show normalized values of the parameters (this means that the obtained value for a particular cell was divided by the value of the CSA-free sample). These and other photovoltaic parameters are presented in the Appendix A in Figure A2. As can be seen, the trend of the dependence of J_sc_ on CSA acid content is similar to the trend of PCE changes, namely, the J_sc_ of samples with a CSA content of up to 2 mg/mL are equal to or higher compared to the reference cell value. Then, the J-V characteristics of a perovskite-based cell with 3 mg/mL CSA revealed about 80% of J_sc_ compared to the perovskite without CSA. Finally, the materials with 4 and 5 mg/mL resulted in devices maintaining about half of the J_sc_ of the reference cell. Regarding series resistance, cells based on perovskites containing up to 2 mg/mL of CSA had similar or slightly higher R_s_ than the reference one. On the other hand, perovskite solar cells with CSA concentrations of 4 and 5 mg/mL showed a relatively high R_s_ (increase of about 50% vs. the reference one).

The deterioration of the cell parameters with higher concentrations of CSA (3, 4, and 5 mg/mL) occurred despite the fact that the perovskite grains were about 50% bigger than in the reference material (Figure 4). PCEs dropped down by more than a factor of two. As it was already discussed above, we observed CSA precipitations on the film surfaces of perovskites with high CSA content. It can therefore be assumed that such precipitates of insulating material (HOMO(eV) −7.165 eV LUMO(eV) 1.157 eV) [21] formed a non-conductive interface (CSA crystallites) in some places between a perovskite layer and HTL (Spiro-OMeTAD). As a consequence, the serial resistance of the devices increased and current density dropped, which significantly reduced the PCE of high-concentration-CSA-based perovskite cells.

### 2.3. Degradation

**Figure 10:**
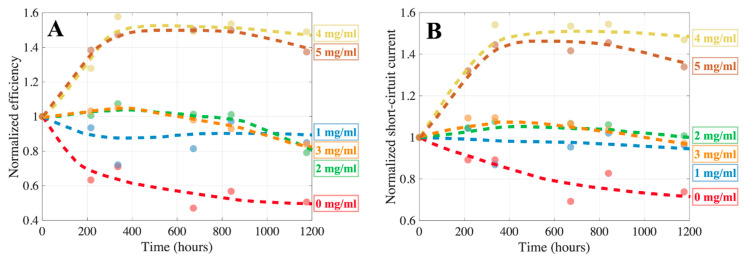
Aging of perovskite solar cells: efficiency (**A**) and short-circuit current (**B**).

The changes of the normalized PCE as a function of time, presented in Figure 10A, indicate different degradation dynamics depending on the concentration of CSA within the perovskite layer.

First, the fastest PCE decay within the set was observed for the reference sample (0 mg/mL), reaching about 0.5 of the initial efficiency after almost 1200 h.

Second, cells with moderate CSA concentrations (1, 2, 3 mg/mL) revealed better stability than the reference (all of the devices end up with about 0.8 of their initial PCEs at the end of the aging experiment). As it was already discussed in the introduction, the perovskites are sensitive to oxygen and moisture, which may diffuse into the perovskite and lead to its gradual decomposition. It has been shown that the increase of the perovskite grain size can significantly reduce oxygen diffusion and considerably slow down the degradation [22]. We observed that a moderate concentration of CSA promotes the growth of large perovskite grains; therefore, these films are supposed to be more stable than the referential material. As a consequence, the devices based on these materials reveal better environmental stability compared to the cells with the pristine perovskite.

**Figure A3:**
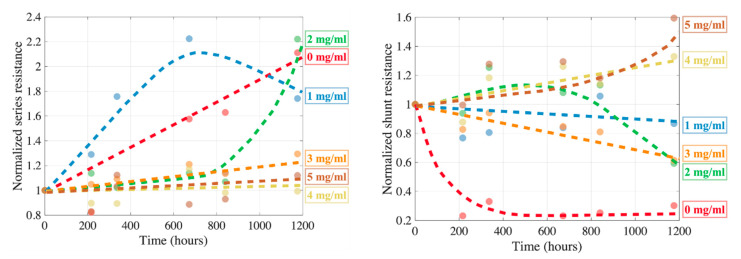
Evolution of series and shunt resistances over time.

Finally, the two cells with highest CSA content (4 and 5 mg/mL) behaved significantly different from the others. Their efficiencies gradually increased, reaching about 1.5 of the initial PCE after 400 h, and then the PCEs saturated at this level. The PCE growth was mainly related to the increase of J_sc_ (Figure 10B). We believe that this increase was caused by interactions of CSA being in form of precipitates with Spiro-OMeTAD. As has been shown, the addition of acids can catalyze the oxidation process of Spiro-OMeTAD in the presence of alkali metal salts [23]. The CSA precipitations were observed on the surface of the perovskite films at relatively high acid concentrations (Figure 3); therefore, we suppose that excess CSA at the interface between the perovskite film and HTL (Spiro-OMeTAD) could migrate to Spiro-OMeTAD, promoting its oxidation and, consequently, improving HTL conductivity. However, it is worth noting that although the relative increase of the PCE was the highest for the cells containing 4 and 5 mg/mL of CSA, the maximum values of absolute PCE of these devices were lower than that containing 2 mg/mL of CSA. The evolution of series and shunt resistances over time is show in Figure A3.

## 3. Materials and Methods

### 3.1. Chemicals

Camphorsulfonic acid (CSA) (≥98.0%), dimethylformamide (DMF), dimethylsulfoxide (DMSO), 1-butanol, acetonitrile, bis(trifluoromethane)sulfonimide lithium salt, 2,2′,7,7′-Tetrakis(N,N-di-p-methoxyphenylamine)-9,9′-spirobifluorene (Spiro-OMeTAD), methylammonium iodide (MAI), and PbI_2_ were acquired from Sigma-Aldrich. Titanium diisopropoxide bis(acetylacetonate) (TAA) and cobalt dopant FK209 TFSI salt were acquired from Merck.

### 3.2. Preparation of Perovskite Thin Films

MAI and PbI_2_ precursors were mixed in DMF/DMSO (4:1 *v*/*v*) with 1:1 molar ratio of MAI and PbI_2_ (1M concentration of each precursor). For the purpose of this study, camphorsulfonic acid (CSA) with different concentrations was added to the perovskite precursor solution (1 to 5 mg/mL of CSA concentration). Then, the mixture was spin-coated at 1000 rpm for 10 s and 4000 rpm for 30 s on glass substrates without stopping in between and subsequently placed on a hotplate at 100 °C for 60 min to convert the perovskite precursor films into MAPbI_3_.

### 3.3. Preparation of Solar Cells

#### 3.3.1. Cleaning of ITO Substrates

In the first step, ITO-coated glass was dipped in DI water containing a Hellmanex soap and was sonicated for 10 min. Successively, the glass slides were sonicated for 10 min in DI water and isopropyl alcohol. Then, the glass slides were dried in filtered compressed nitrogen. In the final step, ITO-coated glass slides were exposed to UV radiation and ozone for 15 min prior to deposition of TiO_2_ layer.

#### 3.3.2. Preparation of ETL

The TiO_2_ layer was deposited by spin coating a 0.15 M TAA solution dispersed in anhydrous 1-butanol and further annealed on a hot plate at 120 °C for 5 min to remove the remaining solvent. In the next step, mesoporous TiO_2_ was spin-coated and annealed at 120 °C again to remove the remaining solvent. Finally, the substrates were annealed for 60 min at 450 °C in a furnace for crystallization of the deposited amorphous TiO_2_.

#### 3.3.3. Preparation of Perovskite Layer

After TiO_2_ preparation, the substrates were transferred into a glovebox with an argon atmosphere. Perovskite layers were deposited analogically to the thin film procedure described above.

#### 3.3.4. Preparation of HTL

The Spiro-OMeTAD solution was prepared by dissolving 85 mg of spiro-OMeTAD in 1 mL of chlorobenzene, 28.8 μL 4-tert- butyl-pyridine, a 20 μL portion of stock solution of 500 mg/mL lithium bis(trifluoromethylsulfonyl) imide in anhydrous acetonitrile, and a 11 μL of cobalt dopant FK209 TFSI salt with a concentration of 300 mg/mL in anhydrous acetonitrile. Such solutions were spin-coated on the perovskite film. Finally, an 80 nm thick Au electrode was deposited via thermal evaporation.

## 4. Conclusions

Studies of a set of perovskite solar cells with a modified active layer by adding CSA acid to the perovskite precursor showed an average improvement of their efficiency by 20% for 1 mg/mL of CSA. A systematic characterization was performed of the influence of CSA acid on the active layer film quality and photovoltaic parameters of the devices as a function of CSA content. It was observed that the higher the CSA content was, the more random the grain orientation within the film was. Additionally, it was found that higher concentrations of CSA led to increases of grain sizes (by 50% compared to the reference with no CSA). However, starting from the perovskite with 3 mg/mL of CSA, precipitations of this acid were observed on the surface of the active layer material. These CSA precipitations formed insulating islands at some places of the interface between the perovskite films and Spiro-OMeTAD within a cell, which resulted in a significant increase in series resistance, thereby reducing the open-circuit current and decreasing the efficiency as a consequence. Aging experiments showed that the cells based on perovskites with moderate concentrations of CSA reveal better environmental stability compared to the reference device. For cells with the highest concentrations of CSA, a gradual increase of PCE over time was observed, which was attributed to CSA migration from its precipitates to Spiro-OMeTAD, promoting Spiro-OMeTAD oxidation, and as a consequence, improving its conductivity.

## Figures and Tables

**Table 1:** Photovoltaic parameters of the perovskite solar cells: PCE—power conversion efficiency; V_oc_—open-circuit voltage; J_sc_—short-circuit current; FF—fill factor; ρ_s_—series resistance; ρ_sh_—shunt resistance.

CSA Content (mg/mL)	Mean PCE (%)	Max PCE (%)	V_oc_ (V)	J_sc_ (mA cm^−2^)	FF (%)	ρ_s_ (Ω cm^2^)	ρ_sh_ (Ω cm^2^)
0	8.74	10.40	0.95	17.65	61.83	6.40	1297.56
1	10.56	11.17	0.98	18.16	62.65	6.57	3880.28
1.3	10.32	11.03	0.98	18.51	60.81	6.84	7026.6
2	9.19	11.73	1.02	17.96	64.34	7.03	5370.4
3	6.34	7.35	0.90	13.80	59.25	8.72	816.52
4	4.11	4.78	0.91	8.64	61.08	8.79	585.32
5	3.89	4.47	0.92	8.15	59.52	10.90	562.24

**Figure A1:**
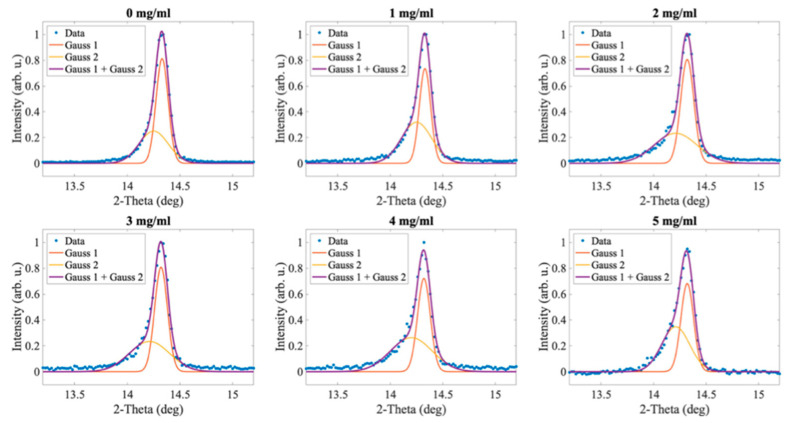
Deconvolution of the XRD peak composed of (110) and (002) reflexes into Gauss curves. Gauss 1—(110) reflex, Gauss 2—(002) reflex.

## Data Availability

Not applicable.

## Appendix A

Table A1 shows mean photovoltaic parameters of fabricated solar cells with CSA.

**Table A1:** Mean photovoltaic parameters of perovskite solar cells. PCE, efficiency; V_oc_, open-circuit voltage; J_sc_, short-circuit current; FF, fill factor; ρ_s_, series resistance; R_sh_, shunt resistance.

CSA Content (mg/mL)	PCE	Voc	Jsc	FF	ρ_s_	ρ_sh_
0	8.74	0.93	16.41	57.14	11.71	359.12
1	10.56	0.98	17.58	61.41	12.29	1222.72
1.3	10.32	0.96	17.58	60.95	11.53	1042.64
2	9.19	0.98	16.25	57.80	12.66	670.35
3	6.34	0.87	12.84	56.63	13.65	577.20
4	4.11	0.91	7.36	61.48	17.36	451.93
5	3.89	0.91	7.19	59.60	17.67	414.53

XRD data reveal a double-peak character of the first (i.e., at the lowest 2-Theta angle) perovskite diffraction peak (Figure A1). It consists of two diffraction lines: (110) and (002). Photovoltaic parameters in a function of CSA content are shown in Figure A2.

Evolution of both series and shunst resistances over time are shown in Figure A3.

## References

[1] Bera S., Saha A., Mondal S., Biswas A., Mallick S., Chatterjee R., Roy S. (2022). Review of Defect Engineering in Perovskites for Photovoltaic Application. Mater. Adv..

[2] Bryant D., Aristidou N., Pont S., Sanchez-Molina I., Chotchunangatchaval T., Wheeler S., Durrant J.R., Haque S.A. (2016). Light and Oxygen Induced Degradation Limits the Operational Stability of Methylammonium Lead Triiodide Perovskite Solar Cells. Energy Environ. Sci..

[3] Yuan H., Debroye E., Janssen K., Naiki H., Steuwe C., Lu G., Moris M., Orgiu E., Uji-I H., De Schryver F. (2016). Degradation of Methylammonium Lead Iodide Perovskite Structures through Light and Electron Beam Driven Ion Migration. J. Phys. Chem. Lett..

[4] Wang D., Wright M., Elumalai N.K., Uddin A. (2016). Stability of Perovskite Solar Cells. Sol. Energy Mater. Sol. Cells.

[5] Shang Y., Fang Z., Hu W., Zuo C., Li B., Li X., Wang M., Ding L., Yang S., Shang Y. (2021). Efficient and Photostable CsPbI_2_Br Solar Cells Realized by Adding PMMA. J. Semicond..

[6] Liu S., Guan Y., Sheng Y., Hu Y., Rong Y., Mei A., Han H. (2020). A Review on Additives for Halide Perovskite Solar Cells. Adv. Energy Mater..

[7] Kim K., Han J., Maruyama S., Balaban M., Jeon I. (2021). Role and Contribution of Polymeric Additives in Perovskite Solar Cells: Crystal Growth Templates and Grain Boundary Passivators. Sol. RRL.

[8] Pan J., Mu C., Li Q., Li W., Ma D., Xu D. (2016). Room-Temperature, Hydrochloride-Assisted, One-Step Deposition for Highly Efficient and Air-Stable Perovskite Solar Cells. Adv. Mater..

[9] Heo J.H., Song D.H., Im S.H. (2014). Planar CH_3_NH_3_PbBr_3_ Hybrid Solar Cells with 10.4% Power Conversion Efficiency, Fabricated by Controlled Crystallization in the Spin-Coating Process. Adv. Mater..

[10] Xu X., Chueh C.C., Yang Z., Rajagopal A., Xu J., Jo S.B., Jen A.K.Y. (2017). Ascorbic Acid as an Effective Antioxidant Additive to Enhance the Efficiency and Stability of Pb/Sn-Based Binary Perovskite Solar Cells. Nano Energy.

[11] Su L., Xiao Y., Han G., Lu L., Li H., Zhu M. (2019). Performance Enhancement of Perovskite Solar Cells Using Trimesic Acid Additive in the Two-Step Solution Method. J. Power Sources.

[12] Han F., Luo J., Ashraf Malik H., Zhao B., Wan Z., Jia C. (2017). A Functional Sulfonic Additive for High Efficiency and Low Hysteresis Perovskite Solar Cells. J. Power Sources.

[13] Kwiatkowska E., Mech W., Wincukiewicz A., Korona K.P., Zarębska K., Kamińska M., Skompska M. (2021). Investigation of Polyaniline Doped with Camphorsulfonic Acid in Chloroform Solution as a Hole Transporting Layer in PTB7: PCBM and Perovskite-Based Solar Cells. Electrochim. Acta.

[14] Liu Y., Zheng X., Fang Y., Zhou Y., Ni Z., Xiao X., Chen S., Huang J. (2021). Ligand Assisted Growth of Perovskite Single Crystals with Low Defect Density. Nat. Commun..

[15] Wang S., Pang S., Chen D., Zhu W., Xi H., Zhang C. (2021). Improving Perovskite Solar Cell Performance by Compositional Engineering via Triple-Mixed Cations. Sol. Energy.

[16] (1S)-(+)-Camphor-10-Sulphonic Acid|3144-16-9. https://www.chemicalbook.com/ChemicalProductProperty_EN_CB0395928.htm.

[17] Ahn N., Son D.Y., Jang I.H., Kang S.M., Choi M., Park N.G. (2015). Highly Reproducible Perovskite Solar Cells with Average Efficiency of 18.3% and Best Efficiency of 19.7% Fabricated via Lewis Base Adduct of Lead(II) Iodide. J. Am. Chem. Soc..

[18] Li L., Chen Y., Liu Z., Chen Q., Wang X., Zhou H. (2016). The Additive Coordination Effect on Hybrids Perovskite Crystallization and High-Performance Solar Cell. Adv. Mater..

[19] Nukunudompanich M., Budiutama G., Suzuki K., Hasegawa K., Ihara M. (2020). Dominant Effect of the Grain Size of the MAPbI3 Perovskite Controlled by the Surface Roughness of TiO2 on the Performance of Perovskite Solar Cells. CrystEngComm.

[20] Kim H.D., Ohkita H., Benten H., Ito S. (2016). Photovoltaic Performance of Perovskite Solar Cells with Different Grain Sizes. Adv. Mater..

[21] Sangeetha P., Mullainathan S., Muthu S., Rajaraman B.R., Saral A., Selvakumari S. (2020). Investigation of Spectroscopic (FT-IR, FT-Raman), Reactive Charge Transfer and Docking Properties of (1S) -(+)-10-Camphorsulfonic Acid by Density Functional Method. Mater. Today Proc..

[22] Aristidou N., Eames C., Sanchez-Molina I., Bu X., Kosco J., Islam M.S., Haque S.A. (2017). Fast Oxygen Diffusion and Iodide Defects Mediate Oxygen-Induced Degradation of Perovskite Solar Cells. Nat. Commun..

[23] Li Z., Tinkham J., Schulz P., Yang M., Kim D.H., Berry J., Sellinger A., Zhu K. (2017). Acid Additives Enhancing the Conductivity of Spiro-OMeTAD Toward High-Efficiency and Hysteresis-Less Planar Perovskite Solar Cells. Adv. Energy Mater..

